# “Splitting the matrix”: intussusceptive angiogenesis meets MT1‐MMP

**DOI:** 10.15252/emmm.201911663

**Published:** 2019-12-20

**Authors:** Gabriela D'Amico, José M Muñoz‐Félix, Ana Rita Pedrosa, Kairbaan M Hodivala‐Dilke

**Affiliations:** ^1^ Barts Cancer Institute John Vane Science Centre Queen Mary University of London London UK

**Keywords:** Digestive System, Immunology, Vascular Biology & Angiogenesis

## Abstract

Pathological angiogenesis contributes to tumour progression as well as to chronic inflammatory diseases. In this issue of *EMBO Molecular Medicine*, Esteban and co‐workers identify endothelial cell MT1‐MMP as a key regulator of intussusceptive angiogenesis (IA) in inflammatory colitis. Thrombospondin 1 (TSP1) cleavage by MT1‐MMP results in the binding of the c‐terminal fragment of TSP1 to αvβ3 integrin, which induces nitric oxide (NO) production, vasodilation and further initiation of IA. This novel control mechanism of inflammatory IA points towards promising new therapeutic targets for inflammatory bowel disease.

## Graphical abstract

Prof. Hodivala‐Dilke and colleagues discuss the findings from Esteban *et al* (in this issue of EMBO Molecular Medicine) that targeting intussusceptive angiogenesis via the TSP1‐nitric oxide axis exhibits therapeutic efficacy in the context of inflammatory bowel disease.

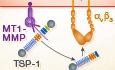

Angiogenesis, the formation and growth of blood vessels, plays an important role in normal development and ischaemic diseases. Increasing evidence supports the existence of different mechanisms of blood vessel growth, including sprouting and intussusceptive angiogenesis (IA). Both sprouting and IA occur under normal physiological and pathological conditions, including tumour growth (De Spiegelaere *et al*, 2012).

Sprouting angiogenesis, where blood vessel endothelial cells locally proliferate, bud and form branching vessels off a main vessel, is an invasive process initiated by multiple matrix metalloproteinases (MMPs). These enzymes degrade extracellular matrix proteins, allowing vascular endothelial cells to invade the tissue and generate vessel sprouts. In contrast, IA is the expansion of the microvasculature through the formation of intraluminal pillars via invagination of the vessel wall into the vascular lumen, ultimately resulting in vessel “splitting”. Blood flow dynamics appear to be crucial in shaping this process, and neither pillar formation nor IA itself appear to be invasive processes (Paku *et al*, 2011). However, little is known about the cellular and molecular control of IA, highlighting the need for improved *in vivo* and *in vitro* models (De Spiegelaere *et al*, 2012).

Sprouting angiogenesis often precedes IA, especially in the tumour setting (Karthik *et al*, 2018). Importantly, since IA is stimulated after initiation of anti‐angiogenic therapy, it constitutes an alternative target to combat vascular disease or resistance to anti‐angiogenic therapy.

In this issue of *EMBO Molecular Medicine*, Esteban *et al* report a previously unrecognised role for endothelial MT1‐MMP in IA (Esteban *et al*, 2020). The expression of the membrane protease MMP family member MT1‐MMP, also known as MMP‐14, is upregulated in angiogenesis and in inflammatory diseases; however, its potential implications in either IA or colitis were unknown.

Here, in a DSS‐induced model of colitis, mice lacking endothelial cell‐MT1‐MMP had reduced number of IA capillary events, resulting in ameliorating colitis symptoms. Using a new whole‐mount imaging method to overcome previous limitations in identifying and analysing IA (Hlushchuk *et al*, 2011; Nowak‐Sliwinska *et al*, 2018), the authors showed that MT1‐MMP expression levels were separately controlled in arterioles and capillaries, leading to a novel bimodal role of action for this enzyme. Constitutive MT1‐MMP expression in arterioles drove NO production, increasing blood flow in the downstream capillary plexus. However, in the capillary y‐junctions, DSS‐induced upregulation of MT1‐MMP drove NO production, which led to endothelial cell remodelling and pillar formation. Although not confirmed in the current study, the authors previously showed that NO regulates MT1‐MMP activity during endothelial cell migration, therefore suggesting that, by a positive feed‐back loop, NO could further reinforce MT1‐MMP upregulation (Genis *et al*, 2007).

Moreover, by combining a Cleavpredict search with *in silico* protein modelling, the authors identified that MT1‐MMP drives TSP1 cleavage, allowing c‐terminal TSP1 fragment release and interaction with CD47/αvβ3 integrin in the induction of NO during IA (Fig 1). This elegant *in silico* analysis could potentially be expanded to aid identification of novel substrates and the subsequent development of cell‐specific strategies.

**Figure 1:**
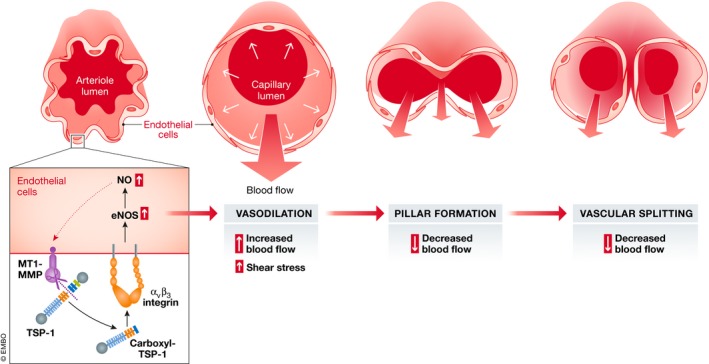
Schematic representation of the proposed model by Esteban *et al* 2020 Endothelial cell MT1‐MMP cleaves TSP1 to generate c‐terminal fragments of TSP1. These in turn bind αvβ3 integrin, leading to NO production. NO production induces vasodilation in arterioles and initiates the process of intussusceptive remodelling. The process of pillar formation and vascular splitting reduces the blood flow in inflamed intestinal mucosa. Targeting this pathway would help control colitis symptoms.

Targeting αvβ3 integrin has been extensively studied in angiogenesis (Robinson & Hodivala‐Dilke, 2011), and some inhibitors such as the RGD‐mimetic Cilengitide underwent clinical trials for cancer treatment. Unfortunately, Cilengitide did not meet the primary endpoints and therefore failed in randomised clinical trials (Demircioglu & Hodivala‐Dilke, 2016). This present study suggests that specifically targeting αvβ3‐TSP1‐c‐terminal fragment interactions may allow IA targeting. Another important finding of this study is the proposed use of serum cleaved TSP‐1 as a biomarker of ulcerative colitis or related Crohn's disease. TSP‐1 was proposed as a biomarker in different pathologies such as pulmonary hypertension and other cardiovascular diseases, and it would be interesting to investigate whether it could be further used as a biomarker in cancers that undergo IA and could benefit from anti‐IA rather than anti‐angiogenic therapies.

While the clinical and translational applications of this work remain to be established, the present results from Arroyo's laboratory (summarised in Fig 1) provide exciting new opportunities for inflammatory disease control via the specific regulation of IA. All the players in this exquisitely balanced pathway of MT1‐MMP‐TSP1‐αvβ3 have been therapeutic targets in the past, suggesting that drug repurposing may be of value in this context. But should we consider endothelial‐specific MT1‐MMP genetic editing as a real option for improved colitis control? Only the future will tell.

## References

[emmm201911663-bib-0001] De Spiegelaere W , Casteleyn C , Van den Broeck W , Plendl J , Bahramsoltani M , Simoens P , Djonov V , Cornillie P (2012) Intussusceptive angiogenesis: a biologically relevant form of angiogenesis. J Vasc Res 49: 390–404 22739226 10.1159/000338278

[emmm201911663-bib-0002] Demircioglu F , Hodivala‐Dilke K (2016) alphavbeta3 Integrin and tumour blood vessels‐learning from the past to shape the future. Curr Opin Cell Biol 42: 121–127 27474973 10.1016/j.ceb.2016.07.008

[emmm201911663-bib-0003] Esteban S , Clemente C , Koziol A , Gonzalo P , Rius C , Martínez F , Linares PM , Chaparro M , Urzainqui A , Andrés V *et al* (2020) Endothelial MT1‐MMP targeting limits intussusceptive angiogenesis and colitis via TSP1‐nitric oxide axis. EMBO Mol Med 12: e10862 10.15252/emmm.201910862 PMC7005619 31793743

[emmm201911663-bib-0004] Genis L , Gonzalo P , Tutor AS , Galvez BG , Martinez‐Ruiz A , Zaragoza C , Lamas S , Tryggvason K , Apte SS , Arroyo AG (2007) Functional interplay between endothelial nitric oxide synthase and membrane type 1 matrix metalloproteinase in migrating endothelial cells. Blood 110: 2916–2923 17606763 10.1182/blood-2007-01-068080 PMC2018672

[emmm201911663-bib-0005] Hlushchuk R , Makanya AN , Djonov V (2011) Escape mechanisms after antiangiogenic treatment, or why are the tumors growing again? Int J Dev Biol 55: 563–567 21858777 10.1387/ijdb.103231rh

[emmm201911663-bib-0006] Karthik S , Djukic T , Kim JD , Zuber B , Makanya A , Odriozola A , Hlushchuk R , Filipovic N , Jin SW , Djonov V (2018) Synergistic interaction of sprouting and intussusceptive angiogenesis during zebrafish caudal vein plexus development. Sci Rep 8: 9840–9854 29959335 10.1038/s41598-018-27791-6 PMC6026200

[emmm201911663-bib-0007] Nowak‐Sliwinska P , Alitalo K , Allen E , Anisimov A , Aplin AC , Auerbach R , Augustin HG , Bates DO , van Beijnum JR , Bender RHF *et al* (2018) Consensus guidelines for the use and interpretation of angiogenesis assays. Angiogenesis 21: 425–532 29766399 10.1007/s10456-018-9613-x PMC6237663

[emmm201911663-bib-0008] Paku S , Dezso K , Bugyik E , Tovari J , Timar J , Nagy P , Laszlo V , Klepetko W , Dome B (2011) A new mechanism for pillar formation during tumor‐induced intussusceptive angiogenesis: inverse sprouting. Am J Pathol 179: 1573–1585 21827961 10.1016/j.ajpath.2011.05.033 PMC3157284

[emmm201911663-bib-0009] Robinson SD , Hodivala‐Dilke KM (2011) The role of beta3‐integrins in tumor angiogenesis: context is everything. Curr Opin Cell Biol 23: 630–637 21565482 10.1016/j.ceb.2011.03.014

